# Modulation of primary motor cortex after experimentally induced and use-dependent plasticity in young and older adults

**DOI:** 10.1007/s00221-025-07107-7

**Published:** 2025-05-30

**Authors:** John Cirillo, Carys R. Ward, Nicholas Gant, Stacey A. Reading, April Ren, Winston D. Byblow

**Affiliations:** 1 Department of Exercise Sciences, The University of Auckland, Auckland, New Zealand; 2 Centre for Brain Research, The University of Auckland, Auckland, New Zealand; 3 Discipline of Physiology, School of Biomedicine, The University of Adelaide, Adelaide, 5005 Australia

**Keywords:** Aging, Transcranial magnetic stimulation, Intracortical inhibition, Paired associative stimulation, Skill acquisition, Cardiorespiratory fitness

## Abstract

The ability to induce plasticity in human primary motor cortex (M1) may be diminished with advancing age. Intracortical inhibition is critical for M1 plasticity and regular participation in physical activity can promote M1 plasticity. This study assessed modulation of M1 excitability and inhibition after paired associative stimulation (PAS) and motor skill acquisition in young and older adults, which also considered the cardiorespiratory fitness of each participant. Thirty-one older (60–88 years) and 20 young (20–33 years) adults were recruited. Electromyographic recordings were obtained from the dominant hand first dorsal interosseous muscle. A sequential visual isometric force task that required index finger abduction was used to investigate motor skill acquisition. Transcranial magnetic stimulation protocols were used to examine corticomotor excitability and short- and long-interval intracortical inhibition (SICI and LICI) before and after each intervention. Corticomotor excitability, normalized to baseline, increased after PAS in young but not older adults, while no age-related differences were observed after skill acquisition. Facilitation of corticomotor excitability after PAS, but not skill acquisition, was positively correlated with cardiorespiratory fitness. SICI decreased after PAS and increased after skill acquisition, with no differences between age groups. LICI increased for young adults and decreased for older adults after PAS, but did not change after skill acquisition. Overall, there was intervention-specific modulation of inhibition, an age-related difference in LICI after PAS, and higher cardiorespiratory fitness was associated with greater corticomotor excitability facilitation after PAS. These findings may help inform future endeavors focused on attenuating age-related declines in brain and motor-cognitive function.

**Supplementary Information:**

The online version contains supplementary material available at 10.1007/s00221-025-07107-7.

## Introduction

Central nervous system deterioration with aging contributes to motor complications, such as movement slowing and diminished performance (Light and Spirduso 1990; Smith et al. 1999). In the human brain, primary motor cortex (M1) is central for controlling movement and important in learning motor skills by continually adapting from experience (i.e., plasticity) (Sanes and Donoghue 2000). Despite motor deficits that emerge in older adults, plasticity remains viable with aging.

Plasticity in the form of long-term potentiation (LTP) can be experimentally induced in human M1 (Ziemann et al. 2008). For example, the protocol of paired associative stimulation (PAS) induces a lasting increase in corticomotor excitability when peripheral nerve stimulation at the wrist precedes transcranial magnetic stimulation (TMS) over M1 by an interval of 25 ms (Stefan et al. 2000, 2002; Rosenkranz and Rothwell 2006). PAS modulates neuronal circuits that are functionally relevant for motor performance and learning (Ziemann et al. 2004; Stefan et al. 2006; Jung and Ziemann 2009). Use-dependent plasticity, a form of motor learning in humans thought to occur through LTP-like mechanisms (Bütefisch et al. 2000; Muellbacher et al. 2002), is often demonstrated to be impaired in older adults (Sawaki et al. 2003). Age-related effects on PAS have not been extensively investigated, but a systematic review stated that older adults appear to be less responsive to LTP-like PAS compared to young participants (Shah et al. 2024). These findings point to a potential age-related decline in LTP-like mechanisms leading to reduced motor cortex plasticity.

The mechanisms underlying diminished plasticity with advancing age are not completely understood. Morphological, neurochemical and neurophysiological changes that accompany healthy aging suggest M1 plasticity differences likely exist between young and older adults, as portrayed in seminal studies (Semmler et al. 2021). However, most studies show that increased corticomotor excitability after skill acquisition is not age-dependent (Berghuis et al. 2017). Corticomotor excitability represents a balance between inhibitory and excitatory inputs within the corticospinal pathway, which may not directly correspond to the magnitude of task performance in an individual (Li Voti et al. 2011; Cirillo et al. 2012). Furthermore, increased corticomotor excitability after executing a task without learning has also been reported (Spampinato and Celnik 2017), although this has not always been a consistent finding (Mooney et al. 2021). While the effects of age are likely to depend on how M1 plasticity is measured and the profile of older adults studied, increased corticomotor excitability after motor skill acquisition throughout adulthood likely reflects an altered functional state of the motor system rather than differences in how the task is performed.

Gamma-aminobutyric acid (GABA)-mediated inhibition is necessary for M1 plasticity. A transient reduction in GABA-mediated inhibition is required for the induction of LTP in M1 (Chen et al. 1994; Hess et al. 1996). In humans, paired-pulse TMS can be used to probe GABA-mediated function within M1 (Chen 2004). With paired-pulse TMS, short-interval intracortical inhibition (SICI) may reflect GABA_A_ receptor activity that mediates phasic inhibitory currents, whereas long-interval intracortical inhibition (LICI) may be a marker of GABA_B_ receptor activity and is typically associated with tonic inhibitory effects (see Ziemann et al. 2015). While conflicting findings in young and older adults preclude whether paired-pulse TMS can discern age-related changes in GABAergic neurotransmission (Bhandari et al. 2016), the ability to modulate GABAergic inhibition may be preserved across the lifespan (Cirillo et al. 2011; Hinder et al. 2011; Mooney et al. 2019). However, most paired-pulse TMS studies induce a posterior–anterior (PA) current in the brain. PA currents preferentially activate early indirect (I) waves (see Di Lazzaro et al. 2012), but late I-wave circuits are most susceptible to the paradigm of SICI (Nakamura et al. 1997; Di Lazzaro et al. 1998b; Hanajima et al. 1998). The direction of the TMS induced current in the brain may recruit different neuronal populations. Anterior–posterior (AP) currents preferentially activate I waves with a later onset latency (Day et al. 1989; Sakai et al. 1997). More SICI has been observed with an AP current than PA (Zoghi et al. 2003; Cirillo and Byblow 2016; Cirillo et al. 2018, 2020; Mooney et al. 2018), indicating that a more robust measure of inhibition may be derived with AP stimulation. Indeed, age-related differences in GABA-mediated inhibition were evident using paired-pulse TMS with AP but not PA stimulation (Sale et al. 2016). Whether AP-compared with PA-induced current in the brain can more effectively detect age-related differences in the modulation of GABA-mediated inhibition after M1 plasticity remains to be elucidated.

Several factors can influence the induction of plasticity, contributing to variability in neurophysiological and behavioral measures. For example, regular participation in physical activity can enhance brain plasticity and improve neurocognitive function (Stillman et al. 2016). While few studies have examined whether regular physical activity influences plasticity in human M1, it has been demonstrated that corticomotor excitability after PAS was greater in physically active young adults compared with sedentary (Cirillo et al. 2009). It was also shown that a 12-week aerobic intervention in healthy sedentary participants modulated MEP threshold and amplitude in addition to improvements in cardiorespiratory fitness (Roeh et al. 2018; Moscatelli et al. 2020). Physical activity levels are commonly reduced with advanced age (Hallal et al. 2012), although more time spent performing physical activity throughout the day has been associated with greater M1 plasticity in older adults (Smith et al. 2021). This association supports literature showing that increased cardiorespiratory fitness can positively affect cognitive plasticity, particularly in the aging brain (Colcombe et al. 2004). Therefore, regular physical exercise that improves cardiorespiratory fitness may positively influence M1 processes, which may account for some inter-individual variability in plasticity responses.

The aim of this study was to assess modulation of corticomotor excitability and GABA-mediated inhibition, using TMS with PA- and AP-induced current, in M1 after PAS and fine motor skill acquisition in young and older adults. Cardiorespiratory fitness of each participant was also considered to reduce inter-individual variability. We hypothesized that: (1) while skill level would be lower for older compared with younger adults both age groups would exhibit successful skill acquisition; (2) corticomotor excitability would increase immediately after PAS for young, but not older, adults, whereas both groups would exhibit increased corticomotor excitability after skill acquisition; (3) the extent of modulation of M1 inhibition (SICI) after a plasticity intervention would be greater with AP compared to PA induced current for both young and older adults.

## Methods

### Participants

Thirty-one older participants (11 females, 20 males; 71 ± 7 years; age range 60–88 years) were recruited. Participants had no known history of peripheral or neurological impairment resulting from neurodegenerative and movement disorders (e.g., Alzheimer’s disease, Parkinson’s disease, Huntington’s disease, Multiple sclerosis, Dystonia, Stroke, Amyotrophic lateral sclerosis) that could affect neurophysiological (single- and paired-pulse TMS) or behavioral measures. Twenty-nine participants were right-handed (median Laterality Quotient of 0.92, range 0.25–1.0) and 2 left-handed (median laterality quotient of − 0.75, range − 0.5 to − 1.0) as assessed by the short version of the Edinburgh handedness questionnaire (Veale 2014). Data from 20 neurologically healthy young controls (9 females, 11 males; 25 ± 3 years; age range 20–33 years) were also collected (19 right-handed, median laterality quotient of 0.82, and 1 left-handed, median laterality quotient of − 0.88). All participants completed a non-invasive brain stimulation safety screening questionnaire based on contraindications reported previously (Rossi et al. 2009, 2021; Ziemann et al. 2015) and a physical activity readiness questionnaire (PAR-Q+) that were screened by a neurologist and clinical exercise physiology practitioner, respectively, before participation. Each participant provided written informed consent and the study was approved by the University of Auckland Human Participants Ethics Committee (UAHPEC 020952).

### Experimental design

**Fig. 1:**
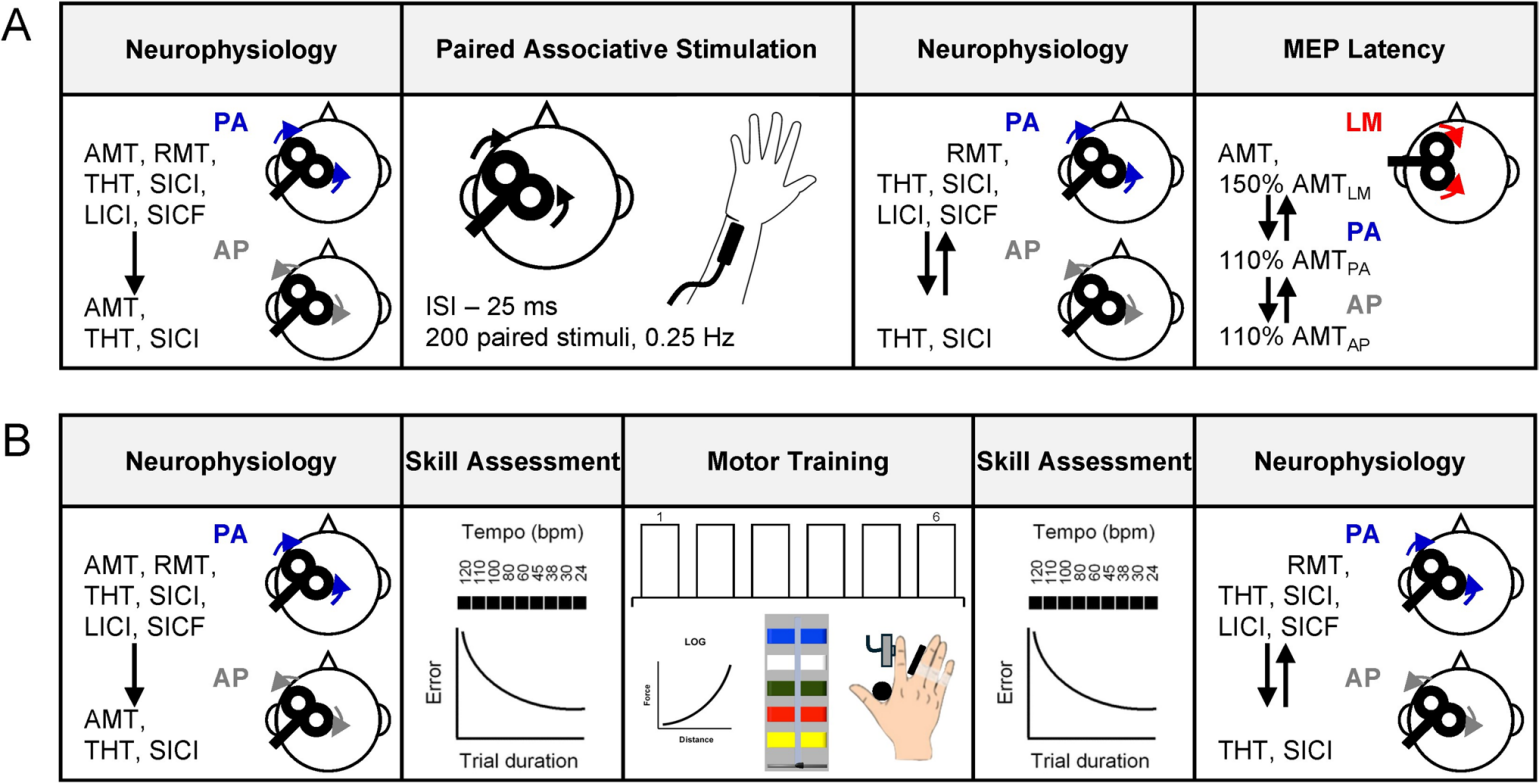
Schematic representation of experimental design. Neurophysiological measures were assessed using single- and paired-pulse transcranial magnetic stimulation (TMS) with posterior-anterior (PA) and anterior–posterior (AP) induced current before and after paired associative stimulation (PAS, **a**) and motor training (**b**). Adaptive threshold-hunting was used to assess active and rest motor thresholds (AMT, RMT), threshold-hunting target (THT), short- and long-interval intracortical inhibition (SICI, LICI), and short-interval intracortical facilitation (SICF). For PAS (**a**), the protocol involved percutaneous electrical stimulation of the ulnar nerve followed by suprathreshold TMS over contralateral primary motor cortex using an interstimulus interval (ISI) of 25 ms, with the intervention consisting of 200 paired stimuli delivered at 0.25 Hz. After the PAS session, motor evoked potential (MEP) onset latencies for PA, AP, and lateromedial (LM) stimulation were also assessed during a low-level isometric voluntary contraction. For motor training (**b**), the task involved navigating the cursor through the sequence red–blue–green–yellow–white by performing isometric index finger abduction against a force transducer. A logarithmic transformation (LOG) was applied to the relationship between applied force and cursor movement of the sequential visual isometric task. The speed–accuracy function was obtained to assess skill before and after motor training by determining error at each trial duration set by an auditory metronome (indicated by tempo in beats per minute; bpm)

Participants completed three sessions in total. For the first and second sessions, two different protocols to induce plasticity, one experimentally with TMS and the other based on motor training, were performed (Fig. 1). Neurophysiology measures were investigated before and after each protocol. In this crossover design the order of the two sessions was pseudorandomized, with both experiments performed in the afternoon and completed at the same time of day for each participant. There was at least 7 days between the first and second session. The third session, performed at least 2 days after the second session, measured cardiorespiratory fitness (e.g., peak oxygen consumption rate) using a graded cardiopulmonary exercise test.

### Neurophysiology

#### Electromyography recordings

Surface electromyography (EMG) was recorded from the dominant first dorsal interosseous (FDI) muscle using 10-mm-diameter Ag–AgCl surface electrodes (Ambu blue sensor paediatric NS, Ballerup, Denmark) placed ~ 2 cm apart in a belly–tendon montage. A ground electrode (3 M Health care, Canada) was placed on the dorsum of the hand. The EMG signals were amplified and band-pass-filtered (10–1000 Hz) using a Bortec AMT-8 (Bortec biomedical Ltd, CA), digitized at 10 kHz with a CED interface system (MICRO1401 mkII, Cambridge electronic design Ltd, UK) and recorded onto a computer for offline analysis using Signal Software (Version 6.05, Cambridge electronic design Ltd, UK).

#### Transcranial magnetic stimulation

Single- and paired-pulse TMS was delivered using a figure-of-eight branding iron coil (70 mm wing diameter), connected to two Magstim 200^2^ magnetic stimulators via a Bistim^2^ unit (Magstim, Whitland, Dyfed, UK). Descending volleys were preferentially activated via early or late onset I-waves by altering current flow through the motor cortex (Day et al. 1989; Werhahn et al. 1994; Sakai et al. 1997; Di Lazzaro et al. 1998a; Hamada et al. 2013). Specifically, posterior–anterior (PA) induced current in the brain preferentially elicits early onset I-waves, whereas anterior–posterior (AP) induced current in the brain preferentially elicits I-waves that have a later onset latency. Each current direction was tested in a single block of trials, with the order of currents pseudorandomized. Optimal scalp position for eliciting a MEP in the contralateral FDI with PA stimulation was marked on the scalp. The same scalp position was used for AP stimulation. TMS was delivered every 4–8 s and the optimal coil position was continually monitored using visual inspection throughout the experiment. During testing participants were seated comfortably with left and right shoulders in a neutral position, the forearm pronated and palm facing down.

#### Motor thresholds

Rest motor threshold (RMT) was defined as the stimulator intensity (expressed relative to maximum stimulator output; MSO) required to elicit a MEP amplitude of at least 50 µV in the relaxed FDI using a freeware program that is based on a maximum-likelihood parameter estimation by sequential testing (PEST) strategy without a priori information (Awiszus 2003; TMS Motor threshold assessment tool; MTAT 2.0). RMT was determined for the PA current direction. Active motor threshold (AMT) was defined as the stimulator intensity required to elicit a MEP amplitude of at least 200 µV during a low-level voluntary precision grip contraction (~ 5% of MVC) using a maximum-likelihood PEST strategy without a priori information (TMS Motor Threshold Assessment Tool; MTAT 2.0). AMT was determined for lateromedial (LM), PA, and AP current directions. For the maximum-likelihood PEST strategy without a priori information, 12 trials are required to estimate the stimulus intensity that elicits the target MEP amplitude within 95% confidence intervals. The duration for a block consisting of 12 TMS trials was ~ 1.5 min.

#### Adaptive threshold-hunting paired-pulse transcranial magnetic stimulation

**Fig. 2:**
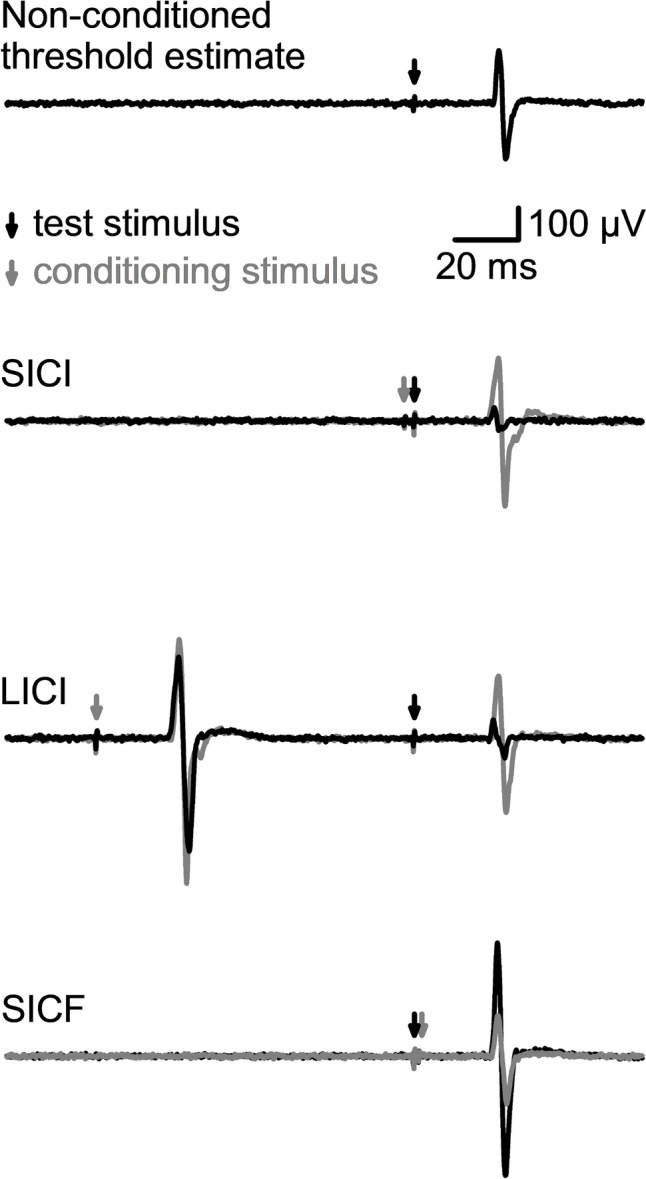
Example electromyography traces depict motor-evoked potentials (MEPs) from the first dorsal interosseus muscle. Traces depict the transcranial magnetic stimulation intensity required to elicit a MEP amplitude of 200 μV to the single-pulse test stimulus (non-conditioned threshold estimate), short-interval intracortical inhibition (SICI), long-interval intracortical inhibition (LICI), and short-interval intracortical facilitation (SICF). For adaptive threshold-hunting, a MEP amplitude greater or less than the target response in the presence of conditioning (black traces) requires a decrease or increase in the test stimulus intensity to restore the target amplitude (gray traces)

Adaptive threshold-hunting involved eliciting a target MEP amplitude of 200 μV (threshold estimate), which represents the middle portion of the linear relationship between the logarithm of the MEP amplitude and the stimulus (Fisher et al. 2002). The adaptive threshold-hunting target was defined as the stimulus intensity required to elicit a MEP amplitude of at least 200 μV in the relaxed FDI (Fig. 2) using a freeware program (TMS Motor threshold assessment Tool; MTAT 2.0). The non-conditioned threshold estimate was determined at the beginning and end of each block of paired-pulse TMS, with the final estimate calculated as the average of these two measurements.

SICI, a measure of phasic inhibitory currents, was investigated for both PA and AP current directions by applying a subthreshold conditioning stimulus (CS) that preceded the test stimulus (TS) by 3 ms (Kujirai et al. 1993). The CS intensity was set to 80% AMT_PA_ for PA stimulation and 80% AMT_AP_ for AP stimulation. In the presence of the CS the TS intensity was increased to reach the adaptive threshold-hunting target MEP amplitude (conditioned threshold estimate; Fig. 2).

LICI and short-interval intracortical facilitation (SICF) were only investigated with the PA current direction. Tonic inhibitory circuits are associated with LICI, whereas SICF provides a marker of glutamatergic transsynaptic activation of corticospinal neurons (Di Lazzaro et al. 2008). For LICI, the CS was defined as the intensity required to elicit a MEP amplitude in the relaxed FDI muscle of at least 1 mV using a maximum-likelihood PEST strategy without a priori information (adaptive 1 mV-hunting). LICI was assessed by delivering this suprathreshold CS 100 ms before the TS (Cirillo and Byblow 2016). For SICF, a subthreshold stimulus (90% RMT_PA_) proceeded the TS by 1.5 ms (Ziemann et al. 1998). Adaptive threshold-hunting was used to determine the TS intensity required to reach the conditioned threshold estimate (MEP amplitude of 200 μV).

All single- and paired-pulse TMS blocks before and after the plasticity intervention were assessed using the maximum-likelihood PEST strategy without a priori information. Each block consisted of 12 trials and was completed in ~ 1.5 min. Before the intervention, 7 blocks of PA stimulation (total of 84 TMS trials) and 4 blocks of AP stimulation (total of 48 TMS trials) were collected (Fig. 1). After the intervention there were 6 blocks of PA stimulation (total of 72 TMS trials) and 3 blocks of AP stimulation (total of 36 TMS trials). The duration of PA stimulation blocks was ~ 10 min, whereas AP stimulation blocks was ~ 5 min. In total, the duration of neurophysiology measures before and after each intervention was 25–30 min.

#### MEP latency

At the end of the experimentally induced plasticity session, MEP latencies were assessed during a low-level voluntary contraction (Wilson et al. 1996; Sakai et al. 1997; Hamada et al. 2013). Stimulation intensities of 110% AMT_PA_ and AMT_AP_ were used to target MEP latency from I waves with an early and late onset, respectively. Lateromedial (LM) stimulation was used to preferentially elicit D waves (Di Lazzaro et al. 1998a). A high LM stimulation intensity (150% AMT_LM_) was used to increase the chances of D-wave recruitment and determine the relative MEP latency onset for PA and AP current directions. Sixteen MEPs were recorded for each current direction and rest periods (30 s) were provided as needed within each block.

### Interventions

#### Paired associative stimulation

PAS was performed as described by Rosenkranz et al. (2006). The PAS protocol consisted of percutaneous electrical stimulation of the ulnar nerve at the wrist of the dominant hand (300% of perceptual threshold) followed by suprathreshold TMS (MEP amplitude of 1 mV) 25 ms later over the optimal scalp position for the index finger. An interval of 25 ms between the peripheral and TMS pulse has been shown to induce an LTP-like MEP increase (Stefan et al. 2000, 2002). The intervention consisted of 200 paired stimuli delivered at 0.25 Hz. To avoid overheating of the TMS coil, monophasic TMS with a PA induced current was delivered using a D70^2^ coil (outer coil diameter 70 mm), connected to a Magstim 200^2^ magnetic stimulator (Magstim, Whitland, Dyfed, UK). The intensity required to elicit a MEP amplitude in the relaxed FDI muscle of at least 1 mV was determined using a maximum-likelihood PEST strategy without a priori information. Electrical stimuli were applied to the ulnar nerve at the wrist using a constant-current stimulator (DS7 A stimulator, Digitimer Co. Ltd, Hertfordshire, UK) with bipolar surface electrodes, separated by 30 mm, and with the cathode proximal. Stimuli were square wave pulses with a pulse width of 200 µs.

The attentional focus of the participant is an important factor influencing PAS effectiveness (Stefan et al. 2004). To quantify attention, participants were instructed to count and report the number of peripheral stimuli they perceived during the PAS intervention (total of 200 stimuli).

#### Skill acquisition task

A sequential visual isometric force task that required index finger abduction was used to investigate motor skill acquisition (Reis et al. 2009; Schambra et al. 2011; Coxon et al. 2014; Statton et al. 2015). Participants sat in front of a computer screen, with the dominant hand positioned palm down on a custom designed platform on the table and their arm abducted at the shoulder and bent at ~ 90° at the elbow. The thumb and all digits other than the index finger were immobilized. The distal interphalangeal joint of the index finger was placed against a MLP-50 force transducer (Transducer Techniques, Temecula, CA). Performing isometric index finger abduction against the transducer vertically displaced a cursor on the screen. The participants were instructed to produce five individual force peaks by moving the cursor to five targets on the screen in a specific color sequence (red–blue–green–yellow–white) and returning to the home position between each color (Coxon et al. 2014). The furthest target was set at 45% of the participant’s maximum voluntary abduction force. A logarithmic transformation was applied to the relationship between applied force and cursor movement.

To assess skill, a speed accuracy function (SAF) was computed by measuring error at fixed execution speeds (Reis et al. 2009). Participants were instructed to generate forces such that the cursor reached the center of each target in time with an auditory metronome beat. The SAF was determined after completing 45 trials, 5 repetitions of 9 different tempos, 24/30/38/45/60/80/100/110/120 bpm, corresponding to approximate trial durations of 12.5/10.0/7.9/6.7/5.0/3.8/3.0/2.7/2.5 s respectively. The SAF was completed before and after training, with the order of tempos randomized for each participant. Evaluating performance across a broad range of speeds and accuracies helps mitigate task-specific adaptation and provides insight into where training may be most effective.

Training of the task was performed by participants at a self-selected pace, with the aim of completing each trial as quickly and as accurately as possible (Reis et al. 2009). Each participant completed 5 blocks of 12 trials (60 trials total), with a 1-min rest period between blocks to avoid fatigue. Visual feedback of mean block skill was provided to participants during the rest periods along with one of two performance-dependent messages: “Well done! Your skill has improved compared with the previous block”; or “Try harder! Your skill has decreased compared with the previous block”.

#### Cardiorespiratory fitness assessment

Participants underwent a cardiorespiratory fitness test on an electronically braked bicycle ergometer (Ergoselect 100, Ergoline, Lindenstrasse 5, 72,475 Bitz, Germany). A linear incremental test protocol with 2-minute stages was used to elicit a maximal rate of oxygen consumption (*V*O_2_ peak). Baseline measures of heart rate (HR), blood pressure (BP) and resting electrocardiography (ECG) were collected during a 5-min rest period seated on the bike. Following the rest period, 5 min of light resistance cycling was used as a warmup prior to completing each stage of increased cycling resistance until reaching volitional fatigue. The cycling resistance for warmup and each test stage were estimated for each participant using their predicted maximal oxygen consumption rate and self-reported time spent in moderate intensity physical activity so that the graded portion of the test could be completed within 8–10 min (Wasserman et al. 2004). HR, BP, and an OMNI 1–10 rating of perceived exertion (RPE) (Robertson et al. 2003) were recorded during warm-up and the final minute of each stage. Exhaled air was collected using a mouthpiece attached to a two-way non-rebreathing valve, secured with headset (Model 2700, Hans Rudolph, Kansas, USA). Expired air and gas composition were measured by metabolic cart containing a pnemotachometer, infrared CO_2_ sensor, and paramagnetic O_2_ detector (TrueOne 2400, ParvoMedics, Utah, USA). Breath by breath data were collected and reported in 15-s epochs. The largest oxygen consumption rate over 15-s during the final exercise stage was used to identify the peak rate of oxygen consumption (*V*O_2_ peak) and this was used as a proxy for the participant’s maximum oxygen consumption rate (*V*O_2_ max).

### Data analysis

#### Neurophysiology

Adaptive threshold-hunting trials contaminated by pre-stimulus EMG activity (root mean squared EMG > 10 µV; 100 ms before stimulation) were rejected online and repeated immediately. Corticomotor excitability was indexed from the mean peak-to-peak amplitude of the 12 MEPs elicited by the suprathreshold CS from the LICI protocol. The relative percent increase or decrease in the TS intensity required to evoke the target MEP amplitude of 200 μV was used to quantify SICI, LICI and SICF as:

$$\begin{aligned}&Inhibition \;or \;facilitation \left( \% \right)\\& = \left( {\frac{{\left( {conditioned\; threshold \;estimate} \right) - \left( {threshold \;estimate} \right)}}{threshold\; estimate}} \right) \times 100\end{aligned}$$

where positive values indicate inhibition and negative values indicate facilitation. More positive values indicate that a higher TS intensity was required to overcome the inhibitory influence of the CS intensity and reach the target MEP amplitude.

MEP latency onset was determined for each trial using a semi-automated method and defined as the time point where rectified EMG signals following TMS exceeded 2SD of the mean background EMG (100 ms before the stimulus artifact). If pre-contraction EMG was detected as latency onset, the cursor was manually adjusted to the first EMG signal associated with the MEP that exceeded mean + 2SD of the background EMG. The MEP latency difference between PA–LM and AP–LM was used as a measure of I-wave recruitment (Hamada et al. 2013).

#### Sequential visual isometric force task

For each trial, an error value was calculated as the sum of differences between the center of each target and the five respective force peaks (Coxon et al. 2014). Skill was quantified for each participant and time point using the following function (Reis et al. 2009):

$$Skill=\frac{1-error}{error\cdot {\text{ln}}{\left(trial \;duration\right)}^{b}}$$

For all participants, the *b* values were calculated at each time point using the Curve Fitting Tool in MATLAB (MathWorks, MA) to account for idiosyncrasies in the profile of the SAF (Saucedo Marquez et al. 2013; Fujiyama et al. 2017; Mooney et al. 2019). The average *b* value for older adults (1.97 ± 1.38) was not significantly different than the control young group (1.53 ± 0.43; *P* = 0.171). For the training trials, skill (hereby referred to as training performance) was quantified using the same equation above, where trial duration was determined as the time between movement onset and the final force peak. Skill values were transformed with a logarithmic function to homogenize the variance (Reis et al. 2009).

### Statistical analysis

Normality was tested using the Shapiro–Wilk test. Non-normal data (corticomotor excitability, AMT, RMT, non-conditioned threshold estimate, and SICI) were logarithmically transformed to satisfy the assumption of normality. Age, *V*O_2_ peak, maximum index finger abduction force, and attention during PAS were analyzed with independent samples *t-tests*.

Independent *t-tests* were performed on baseline neurophysiology measures of young and older adults for both PAS and skill acquisition sessions. Linear mixed model analysis with fixed effects of AGE (Older, Young) and TIME (before, after) was performed on corticomotor excitability, RMT, LICI, and SICF for both the PAS and skill acquisition interventions. For the non-conditioned threshold estimate and SICI, a linear mixed model analysis with the fixed effects of AGE, TIME, and CURRENT DIRECTION (PA, AP) was also performed for both interventions. One-sample *t-tests* (hypothesized mean = 0) were performed for SICI_PA_, SICI_AP_, LICI, and SICF to confirm the presence of inhibition and facilitation. MEP amplitude is inherently variable (Spampinato et al. 2023) and, to account for individual differences in baseline corticomotor excitability, pre-planned one-sample *t-tests* (hypothesized mean = 100) were also performed for young and older adults to assess within-subject modulation of corticomotor excitability normalized to baseline values:

$$\begin{aligned}&MEP\, \left(\% Baseline\right)\\&= (post-intervention / pre-intervention) \times 100\end{aligned}$$

For the skill acquisition session, linear mixed model analysis with fixed effects of AGE and TIME was performed on SAF assessment to investigate the effect of training. Training performance was also assessed using a linear mixed model analysis with fixed effects of AGE and BLOCK (1, 2, 3, 4, 5). For AMT, a linear mixed model analysis with fixed effects of AGE and CURRENT DIRECTION (PA, AP) was performed.

For the PAS session, linear mixed model analysis with fixed effects of AGE and CURRENT DIRECTION (LM, PA, AP) was performed on AMT and MEP latency. A linear mixed model analysis with fixed effects of AGE and I-WAVE RECRUITMENT (PA–LM, AP–LM) was also performed.

A Spearman correlation analysis (two-tailed) was used to investigate whether there was a relationship between the modulation of corticomotor excitability (MEP (% Baseline)) after PAS and skill acquisition. Spearman correlation analyses were also used to investigate the relationship between modulation of corticomotor excitability after skill acquisition and behavioral outcomes of SAF (i.e., (post-skill/pre-skill) × 100) and training performance (i.e., (B5/B1) × 100). Additional Spearman correlation analyses were used to investigate the relationship between cardiorespiratory fitness and behavioral outcomes, and modulation of corticomotor excitability after PAS and skill acquisition. Separate correlation analyses for each age group were also performed (Supplementary Table 1). For PAS, the relationship between modulation of corticomotor excitability and PA MEP latency was investigated using Spearman correlation analyses (Supplementary Table 2). Regression modelling to determine predictors of age and cardiorespiratory fitness was also examined for baseline skill and task performance.

For all linear mixed effects model analyses, FITNESS (*V*O_2_ peak) was added as a covariate and PARTICIPANT was included as a random effect. Linear mixed effects models with and without the covariate were compared to examine the contribution of fitness to improving the model fit. Results from reduced but best fit models are presented. Post hoc analyses and correlations were corrected using the Hochberg method (Hochberg 1988). The significance level was set at *P* < 0.05 and group data are presented as mean ± SD in the text.

## Results

No adverse events were reported. Two older participants declined to participate in neurophysiology assessment after the first experimental session. As a result, 29 older adults completed the skill acquisition session. Age, cardiorespiratory fitness, maximal index finger abduction force, and attention to the PAS intervention are displayed in Table 1.

**Table 1:** Description of participant characteristics

	Young (N = 20)	Older (N = 31)	*P* value
Age (years)	25.20 (3.38)	71.58 (7.21)	< 0.001
Sex	11 M, 9 F	19 M, 12 F	
*V*O_2_ peak (mL/kg/min)	43.48 (10.68)	27.28 (10.08)	< 0.001
MVC (N)	30.98 (8.70)	30.73 (10.00)	0.930
Attention (total of 200)	197.35 (5.73)	191.74 (21.96)	0.271

*V*O_2_* peak* Maximal rate of oxygen consumption, *MVC* Index finger maximum voluntary contraction

Values are mean (SD)

### MEP latency

MEP latencies and I-wave recruitment are reported in Table 2. A reliable MEP with LM stimulation was not obtained in one older adult. Therefore, MEP latencies and I-wave recruitment were completed for 30 older adults. There was a main effect of Age (F_1_,_47_ = 14.453; *P* < 0.001; ${\eta }_{p}^{2}$ = 0.235), such that MEP latencies were longer for older adults compared with young. There was also a main effect of Current direction (F_2_,_96_ = 253.055; *P* < 0.001; ${\eta }_{p}^{2}$ = 0.841; Table 1). Post hoc testing revealed that PA MEP latency was longer compared with LM (*t*_*49*_ = 10.124, *P*_*adj*_ < 0.001, Cohen’s d = 1.43), and AP MEP latency was longer than LM (*t*_*49*_ = 19.184, *P*_*adj*_ < 0.001, Cohen’s d = 2.71) and PA (*t*_*49*_ = 16.599, *P*_*adj*_ < 0.001, Cohen’s d = 2.35). There was no Age × Current direction interaction (F_2_,_96_ = 0.339; *P* = 0.713; ${\eta }_{p}^{2}$ = 0.007). As expected, I-wave Recruitment (F_1_,_48_ = 270.425; *P* < 0.001; ${\eta }_{p}^{2}$ = 0.849) with a later onset was more indicative with AP-LM current (Young = 4.10 ± 0.73 ms, Older 3.86 ± 1.80 ms) than PA–LM (Young = 1.70 ± 0.63 ms, Older 1.73 ± 1.48 ms). There was no main effect of Age (F_1_,_47_ = 0.084; *P* = 0.773; ${\eta }_{p}^{2}$ = 0.002) or Age × Current direction interaction (F_1_,_48_ = 0.930; *P* = 0.340; ${\eta }_{p}^{2}$ = 0.019).

**Table 2:** Group TMS thresholds and latencies for different current directions

	TMS current direction			*P* value
	LM	PA	AP	
AMT (% MSO)				< 0.001
Young (N = 20)	44.19 (9.26)	36.58 (5.27)	52.49 (8.00)	
Older (N = 31)	55.54 (14.36)	40.89 (6.60)	54.92 (11.48)	
MEP onset latency (ms)				< 0.001
Young (N = 20)	22.02 (2.26)	23.72 (2.41)	26.12 (2.29)	
Older (N = 30)	23.63 (2.66)	25.34 (2.40)	27.43 (2.43)	

*TMS* Transcranial magnetic stimulation, *LM* Lateromedial; *PA* Posterior–anterior, *AP* Anterior–posterior, *AMT* FDI active motor threshold, *MEP* FDI Motor evoked potential, *MSO* Maximum stimulator output

Values are mean (SD)

### Paired associative stimulation (PAS)

Baseline neurophysiology measures were not different between young and older adults (all *P*_*adj*_ > 0.122). One-sample *t-tests* revealed that SICI_PA_, SICI_AP_, LICI, and SICF (all *P*_*adj*_ < 0.001) were present at baseline.

Active motor thresholds are reported in Table 2 and results of the linear mixed model analyses for PAS are displayed in Table 3. For young adults, AMT_LM_ was lower than AMT_AP_ (*t*_*19*_ = − 5.093, *P*_*adj*_ < 0.001, Cohen’s d = − 1.14), and AMT_PA_ was lower than AMT_LM_ (*t*_*19*_ = − 5.348, *P*_*adj*_ < 0.001, Cohen’s d = − 1.20) and AMT_AP_ (*t*_*19*_ = − 11.869, *P*_*adj*_ < 0.001, Cohen’s d = − 2.65). For older adults, AMT_PA_ was lower than AMT_LM_ (*t*_*30*_ = − 7.593, *P*_*adj*_ < 0.001, Cohen’s d = − 1.36) and AMT_AP_ (*t*_*30*_ = − 12.115, *P*_*adj*_ < 0.001, Cohen’s d = − 2.18), but there was no difference between AMT_LM_ and AMT_AP_ (*t*_*30*_ = 0.041, *P*_*adj*_ = 0.999, Cohen’s d < 0.01). There was also a lower AMT_LM_ for young participants compared with older (*t*_*49*_ = − 3.303, *P*_*adj*_ = 0.008, Cohen’s d = − 0.95), but not for AMT_PA_ (*t*_*49*_ = − 2.415, *P*_*adj*_ = 0.078, Cohen’s d = − 0.69) and AMT_AP_ (*t*_*49*_ = − 0.701, *P*_*adj*_ = 0.930, Cohen’s d = − 0.20).

**Table 3:** Statistical results of linear mixed model analyses for paired associative stimulation

PAS	Model term	*df*	*F*	*P*	${\eta }_{p}^{2}$
*Neurophysiology*					
AMT	Age	1,48.0	1.820	0.184	0.037
	Current direction	2,98.0	92.361	< 0.001	0.653
	Age × current direction	2,98.0	7.169	0.001	0.128
RMT	Age	1,48.0	0.877	0.354	0.018
	Time	1,49.0	0.001	0.970	< 0.001
	Age × time	1,49.0	1.982	0.166	0.039
Corticomotor excitability	Age	1,49.0	9.800	0.003	0.167
	Time	1,49.0	0.376	0.543	0.008
	Age × time	1,49.0	2.264	0.139	0.044
LICI	Age	1,49.0	7.425	0.009	0.132
	Time	1,49.0	0.685	0.412	0.014
	Age × time	1,49.0	14.086	< 0.001	0.223
SICF	Age	1,49.0	2.007	0.163	0.039
	Time	1,49.0	5.633	0.022	0.103
	Age × time	1,49.0	0.356	0.466	0.007
Non-conditioned threshold estimate	Age	1,46.2	1.141	0.291	0.024
	Time	1,45.5	2.236	0.142	0.047
	Current direction	1,42.0	556.411	< 0.001	0.930
	Age × time	1,45.5	< 0.001	0.993	< 0.001
	Age × current direction	1,42.0	0.277	0.602	0.007
	Time × current direction	1,44.3	2.495	0.121	0.053
	Age × time × current direction	1,44.3	2.304	0.136	0.049
SICI	Age	1,45.5	< 0.001	0.998	< 0.001
	Time	1,78.7	2.048	0.156	0.025
	Current direction	1,43.0	29.962	< 0.001	0.411
	Age × time	1,78.7	0.648	0.423	0.008
	Age × current direction	1,43.0	0.077	0.782	0.002
	Time × current direction	1,91.8	5.817	0.018	0.060
	Age × time × current direction	1,91.8	0.213	0.645	0.002

**Fig. 3:**
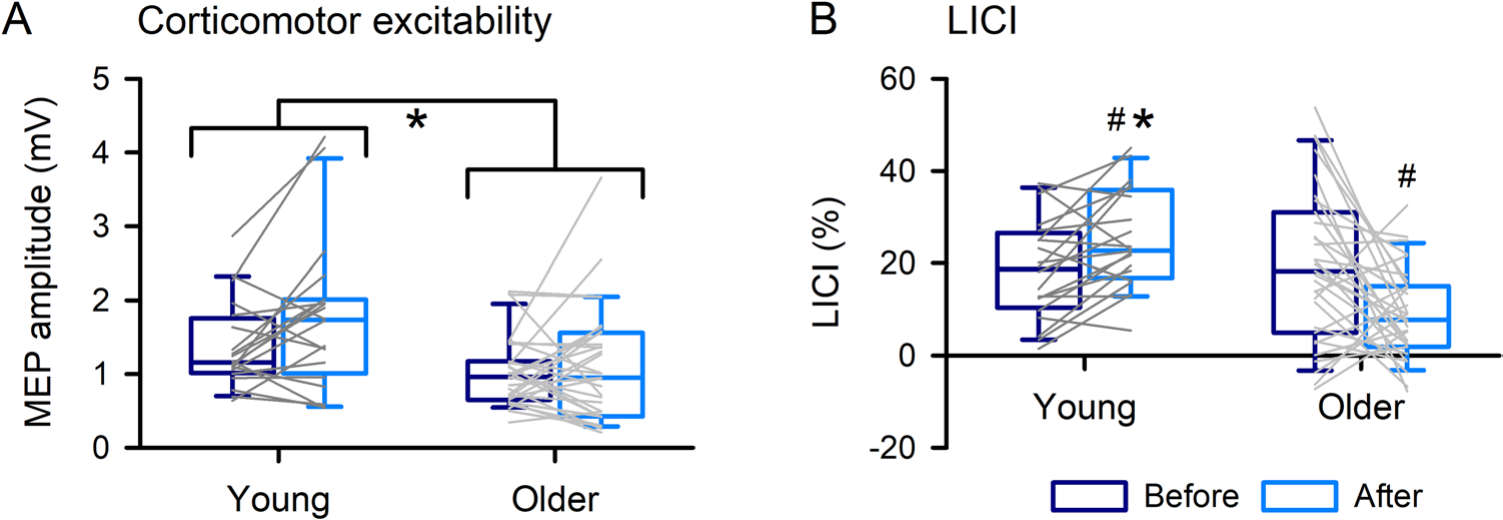
Neurophysiological measures of corticomotor excitability (**a**) and long-interval intracortical inhibition (LICI, **b**), obtained using only posterior-anterior current direction, before and after paired associative stimulation (PAS). LICI increased and decreased after PAS for young and older adults respectively, which was different between groups (**b**). Boxes, 25 th and 75 th percentiles; whiskers, 10 th and 90 th percentiles. Lines match individual data points for each young (dark grey) and older (light grey) adult before and after PAS. *P < 0.05, compared with older; ^#^*P* < 0.05, compared with before PAS

Corticomotor excitability and LICI before and after PAS in young and older adults are shown in Fig. 3. For normalized corticomotor excitability data, one-sample *t-tests* showed that MEP amplitude increased after PAS in young adults (131.06 ± 56.02%;* t*_*19*_ = 2.14, *P*_*adj*_ = 0.046, Cohen’s d = 0.48) but not older (112.37 ± 69.20%%;* t*_*30*_ = 0.21, *P*_*adj*_ = 0.834, Cohen’s d = 0.04). For LICI, there was no difference between young (18.70 ± 10.92%) and older (19.31 ± 16.69%) adults before PAS (*t*_*49*_ = −0.143, *P*_*adj*_ = 0.987, Cohen’s d = −0.04), but LICI was greater in young (25.26 ± 10.84%) compared with older (9.04 ± 9.83%) adults after PAS (*t*_*49*_ = 5.523, *P*_*adj*_ < 0.001, Cohen’s d = 1.58). For young adults LICI increased after PAS (*t*_*19*_ = 3.103, *P*_*adj*_ = 0.012, Cohen’s d = 0.69), whereas LICI decreased after PAS for older adults (*t*_*30*_ = −3.089, *P*_*adj*_ = 0.008, Cohen’s d = −0.56).

**Fig. 4:**
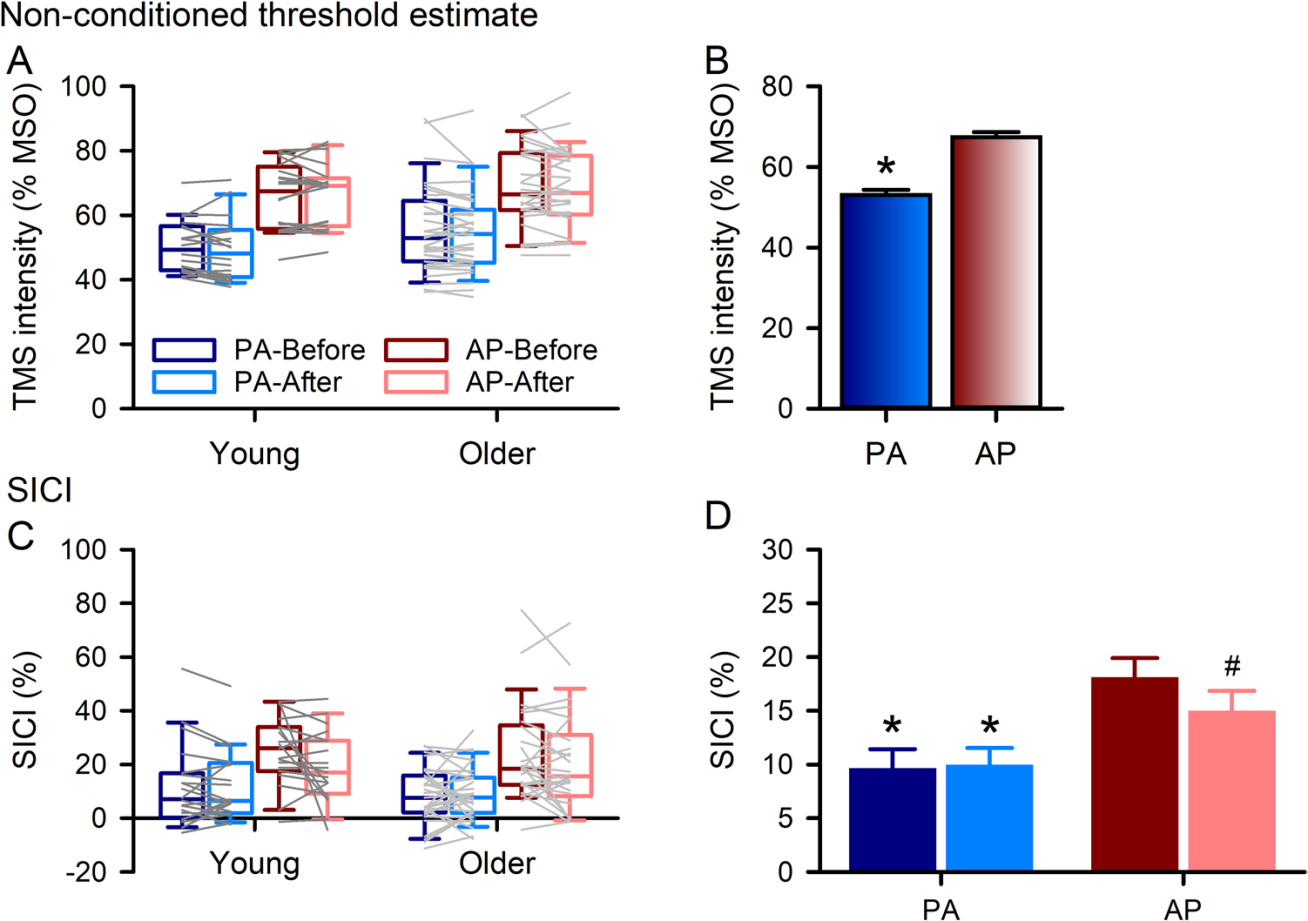
Neurophysiological measures obtained using both posterior-anterior (PA) and anterior–posterior (AP) current directions before and after paired associative stimulation (PAS). Threshold estimate values for adaptive threshold-hunting (target amplitude of 200 μV, **a**) required a higher stimulation intensity for AP current direction than PA (**b**, data collapsed across age groups and time). Short-interval intracortical inhibition (SICI, **c**) was greater using AP stimulation than PA before and after PAS (**d**, data collapsed across age groups), and SICI decreased after PAS for AP stimulation (**d**). Boxes, 25 th and 75 th percentiles; whiskers, 10 th and 90 th percentiles (**a, c**). Lines match individual data points for each young (dark grey) and older (light grey) adult before and after PAS for each current direction (**a, c**). Data presented as mean ± SE (**b, d**). *P < 0.05, compared with AP; ^#^*P* < 0.05, compared with before PAS

Figure 4 shows the PA and AP TMS intensity for the non-conditioned threshold estimate and SICI before and after PAS in young and older adults. A higher stimulation intensity was required to elicit the non-conditioned threshold for AP stimulation than PA (Fig. 4b). For SICI (Fig. 4d), the amount of inhibition was greater for AP stimulation than PA before (*t*_*44*_ = − 7.157, *P*_*adj*_ < 0.001, Cohen’s d = − 1.07) and after (*t*_*44*_ = − 3.806, *P*_*adj*_ < 0.001, Cohen’s d = − 0.57) PAS. SICI was not different for PA stimulation before (Young = 11.79 ± 15.37%, Older 8.32 ± 10.28%) and after (Young = 11.49 ± 12.75%, Older 9.04 ± 9.83%) PAS (*t*_*50*_ = − 0.784, *P*_*adj*_ = 0.683, Cohen’s d = − 0.11), but SICI decreased after PAS (Young = 19.13 ± 13.21%, Older 20.70 ± 18.64%) compared with before (Young = 25.20 ± 12.69%, Older 23.39 ± 17.92%) for AP stimulation (*t*_*44*_ = 2.377, *P*_*adj*_ = 0.044, Cohen’s d = 0.35).

### Skill acquisition—behavioral

**Fig. 5:**
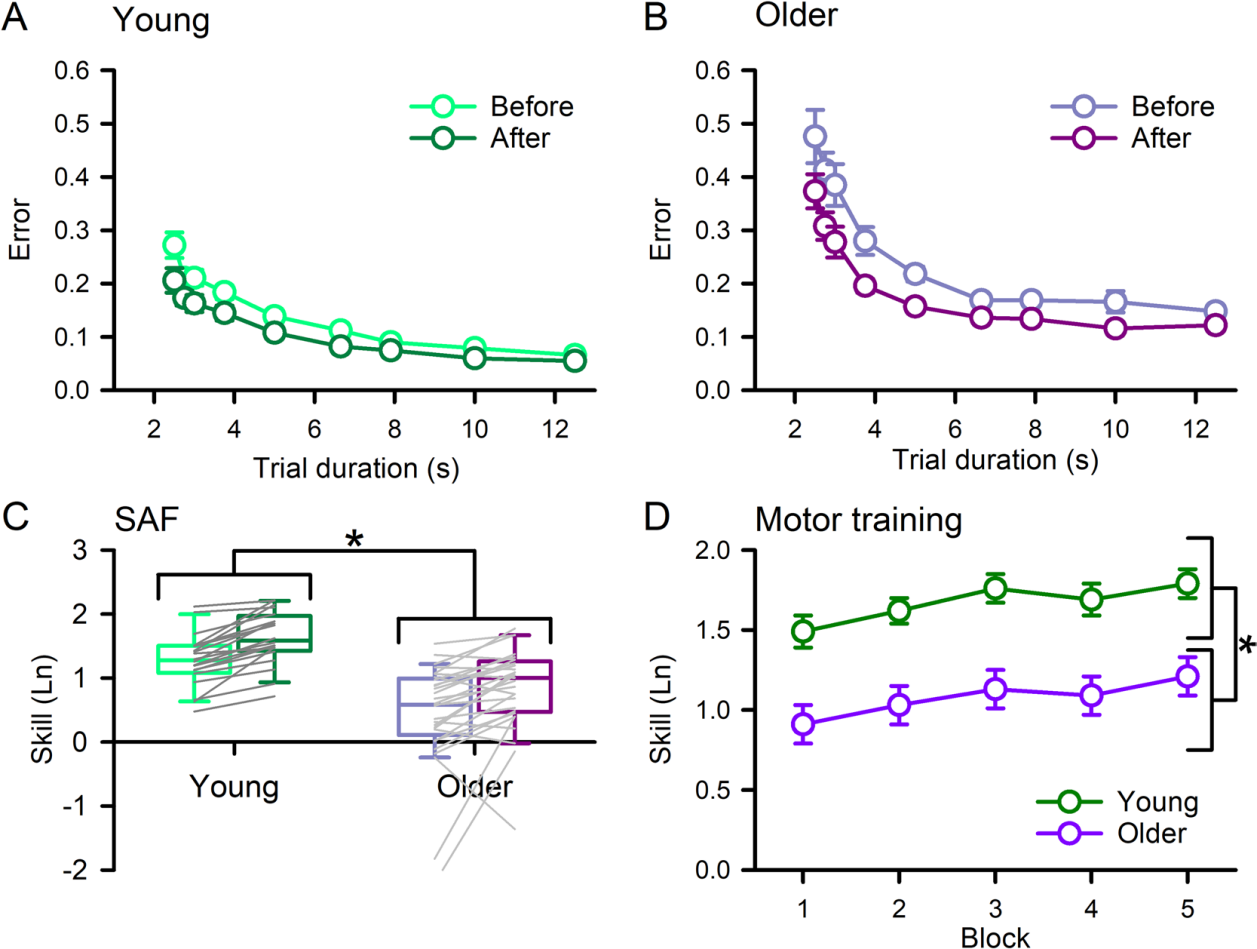
Sequential visual isometric force task. Speed-accuracy function (SAF) data obtained before and after motor training in Young (**a**) and Older (**b**) adults. SAF skill was better in young adults compared with older and improved after motor training (**c**). Training performance was better in young adults compared with older and improved throughout training (**d**). Data presented as mean ± SE (**a**, **b**, **d**). Boxes, 25 th and 75 th percentiles; whiskers, 10 th and 90 th percentiles (**c**). *P < 0.05, compared with older

Behavioral results from the skill acquisition linear mixed model analyses are displayed in Table 4. The SAFs determined before and after motor training for young and older adults are shown in Fig. 5a–c. Skill was better in young adults (1.960 ± 0.281) compared with older (1.583 ± 0.253) and skill improved after motor training (Before = 1.652 ± 0.293, After = 1.822 ± 0.331). Training performance (Fig. 5d) was higher in young adults (2.076 ± 0.290) compared with older (1.728 ± 0.296). Improvement in training performance was greater than B1 (1.771 ± 0.315) for B2 (1.840 ± 0.313; *t*_*48*_ = − 3.172, *P*_*adj*_ = 0.015 Cohen’s d = − 0.45), B3 (1.915 ± 0.363; *t*_*48*_ = − 6.378, *P*_*adj*_ < 0.001 Cohen’s d = − 0.91), B4 (1.886 ± 0.354; *t*_*48*_ = − 4.642, *P*_*adj*_ < 0.001 Cohen’s d = − 0.66), and B5 (1.937 ± 0.336; *t*_*48*_ = − 7.211, *P*_*adj*_ < 0.001 Cohen’s d = − 1.03). Training performance improvement was also greater than B2 for B3 (*t*_*48*_ = − 3.546, *P*_*adj*_ = 0.004 Cohen’s d = − 0.51) and B5 (*t*_*48*_ = − 4.107, *P*_*adj*_ < 0.001 Cohen’s d = − 0.59), and between B4 and B5 (*t*_*48*_ = − 2.623, *P*_*adj*_ = 0.047 Cohen’s d = − 0.37). All other comparisons were not significant (all *P*_*adj*_ > 0.111).

**Table 4:** Statistical results of linear mixed model analyses for skill acquisition

Skill acquisition	Model term	*df*	*F*	*P*	${\eta }_{p}^{2}$
*Behavioral*					
SAF	Age	1,47.0	28.861	< 0.001	0.380
	Time	1,47.0	1.652	0.331	0.034
	Age × time	1,47.0	1.051	0.311	0.022
Training performance	Age	1,46.0	12.978	< 0.001	0.220
	Block	4,188.0	19.410	< 0.001	0.292
	Age × block	4,188.0	0.721	0.578	0.015
*Neurophysiology*					
AMT	Age	1,46.0	3.662	0.062	0.074
	Current direction	1,47.0	320.129	< 0.001	0.872
	Age × current direction	1,47.0	2.701	0.511	0.054
RMT	Age	1,46.0	3.018	0.089	0.062
	Time	1,47.0	0.440	0.511	0.009
	Age × time	1,47.0	1.106	0.298	0.023
Corticomotor excitability	Age	1,47.0	0.119	0.732	0.003
	Time	1,47.0	3.147	0.083	0.063
	Age × time	1,47.0	0.001	0.971	< 0.001
LICI	Age	1,47.0	0.002	0.964	< 0.001
	Time	1,47.0	0.003	0.958	< 0.001
	Age × time	1,47.0	1.038	0.314	0.022
SICF	Age	1,47.0	0.116	0.685	0.002
	Time	1,47.0	0.005	0.945	< 0.001
	Age × time	1,47.0	0.291	0.592	0.006
Non-conditioned threshold estimate	Age	1,44.4	2.000	0.164	0.043
	Time	1,88.4	6.311	0.014	0.067
	Current direction	1,40.3	468.730	< 0.001	0.921
	Age × time	1,88.4	0.267	0.607	0.003
	Age × current direction	1,40.3	1.025	0.317	0.025
	Time × current direction	1,89.6	0.253	0.616	0.003
	Age × time × current direction	1,89.6	0.008	0.930	< 0.001
SICI	Age	1,42.0	0.773	0.384	0.018
	Time	1,85.2	4.415	0.039	0.049
	Current direction	1,40.2	28.174	< 0.001	0.412
	Age × time	1,85.2	0.155	0.695	0.002
	Age × current direction	1,40.2	0.041	0.840	0.001
	Time × current direction	1,88.0	1.459	0.230	0.016
	Age × time × current direction	1,88.0	0.320	0.573	0.004

### Skill acquisition—neurophysiology

Baseline neurophysiology measures did not differ between young and older adults (all *P*_*adj*_ > 0.394). One-sample *t-tests* revealed that SICI_PA_, SICI_AP_, LICI, and SICF (all *P*_*adj*_ < 0.001) were present at baseline.

Results of the linear mixed model analyses for skill acquisition are displayed in Table 4. For AMT, a higher stimulation intensity was required for AP stimulation (Young = 52.49 ± 8.00% MSO, Older 54.92 ± 11.48% MSO) than PA (Young = 36.58 ± 5.27% MSO, Older 40.89 ± 6.60% MSO).

**Fig. 6:**
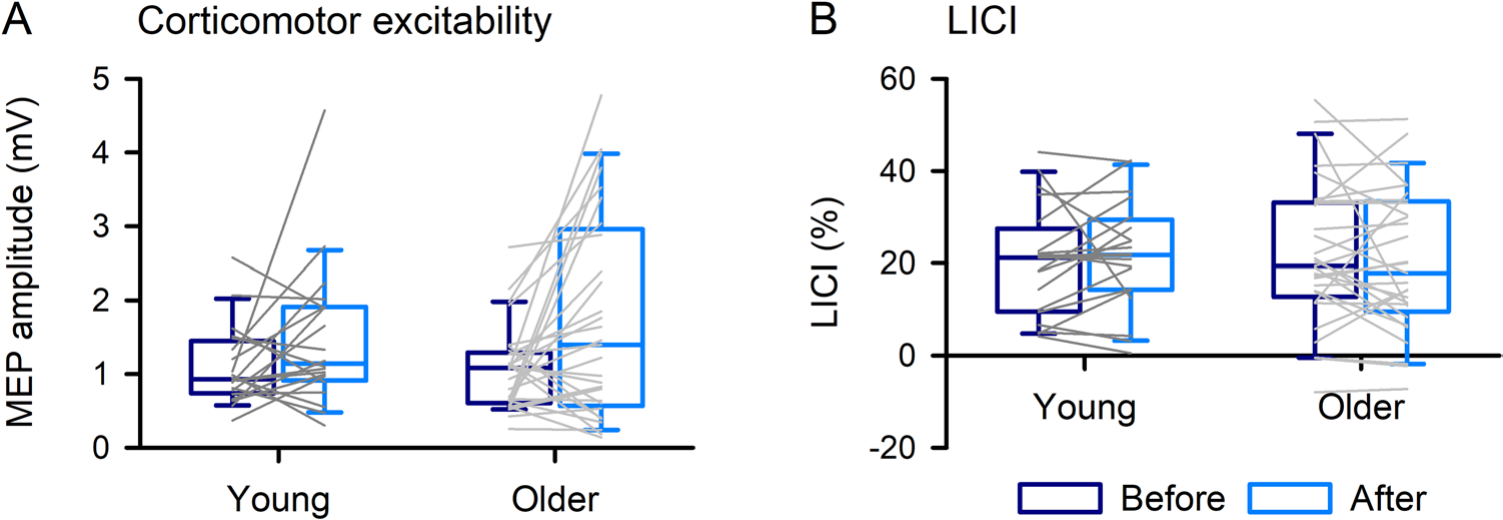
Neurophysiological measures of corticomotor excitability (**a**) and long-interval intracortical inhibition (LICI, **b**), obtained using only posterior–anterior current direction, before and after motor training. Boxes, 25 th and 75 th percentiles; whiskers, 10 th and 90 th percentiles. Lines match individual data points for each young (dark grey) and older (light grey) adult before and after motor training

Corticomotor excitability and LICI before and after skill acquisition in young and older adults are shown in Fig. 6. For normalized corticomotor excitability data, one-sample *t-tests* showed a trend for increased MEP amplitude in young adults (153.87 ± 106.30%;* t*_*19*_ = 1.95, *P*_*adj*_ = 0.067 Cohen’s d = 0.44) and an increase in MEP amplitude for older adults (185.43 ± 180.75%;* t*_*48*_ = 2.91, *t*_*28*_ = 2.21, *P*_*adj*_ = 0.036, Cohen’s d = 0.41). There was no modulation of LICI.

**Fig. 7:**
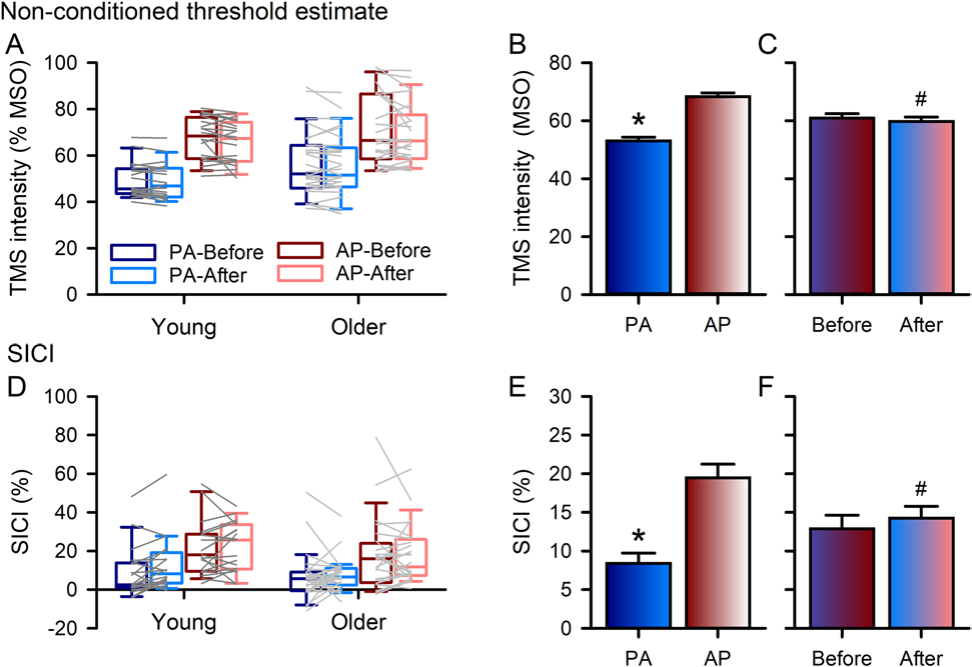
Neurophysiological measures obtained using both posterior-anterior (PA) and anterior–posterior (AP) current directions before and after motor training. Threshold estimate values for adaptive threshold-hunting (target amplitude of 200 μV, **a**) required a higher stimulation intensity for AP current direction than PA (**b**, data collapsed across age groups and time) and decreased after motor training (**c**, data collapsed across age groups and current direction). Short-interval intracortical inhibition (SICI, **d**) was greater using AP stimulation than PA (**e**, data collapsed across age groups and time) and increased after motor training (**f**, data collapsed across age groups and current direction). Boxes, 25 th and 75 th percentiles; whiskers, 10 th and 90 th percentiles (**a, d**). Lines match individual data points for each young (dark grey) and older (light grey) adult before and after motor training for each current direction (**a, d**). Data presented as mean ± SE (**b, c, e, f**). *P < 0.05, compared with AP; ^#^*P* < 0.05, compared with before motor training

Figure 7 shows the PA and AP TMS intensity for the non-conditioned threshold estimate and SICI before and after skill acquisition in young and older adults. Consistent with PAS results, a higher stimulation intensity to elicit the non-conditioned threshold was required for AP stimulation than PA (Fig. 7b). However, a lower stimulation intensity was required to obtain the non-conditioned threshold estimate after skill acquisition than before (Fig. 7c). For SICI, the amount of inhibition was greater for AP stimulation than PA (Fig. 7e) and there was more SICI after skill acquisition than before (Fig. 7f).

### Correlation analyses

**Fig. 8:**
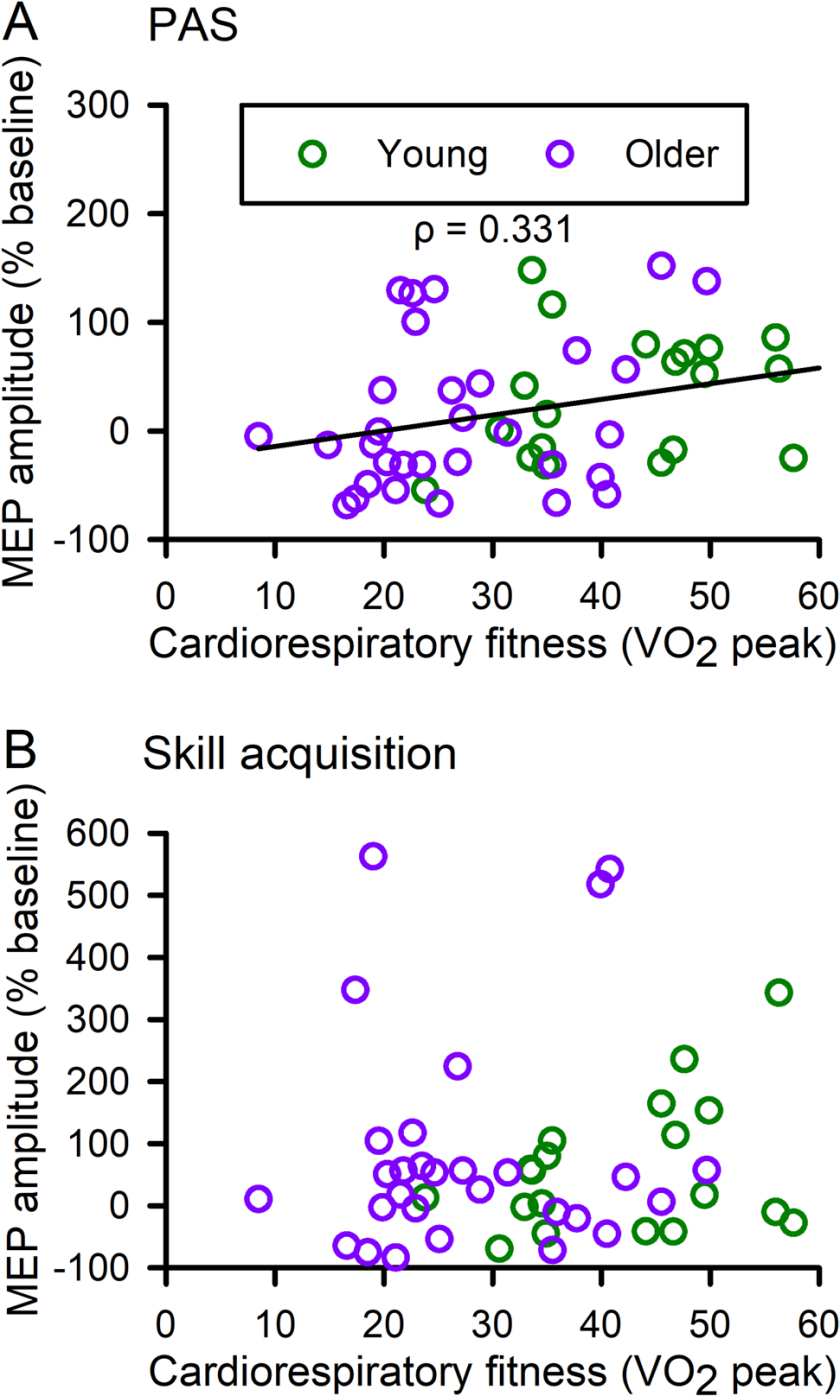
Correlation analyses. Correlations between cardiorespiratory fitness (maximal rate of oxygen consumption, *V*O_2_ peak) and modulation of corticomotor excitability (motor evoked potential amplitude normalized to baseline) after paired associative stimulation (PAS, **a**) and motor training (**b**)

There was no association between modulation of corticomotor excitability after PAS compared to that after skill acquisition (ρ = 0.237, *P*_*adj*_ = 0.194). There was also no association between behavioral outcomes and modulation of corticomotor excitability (SAF ρ = − 0.217, *P*_*adj*_ = 0.250; training performance ρ = 0.071, *P*_*adj*_ = 0.861) or cardiorespiratory fitness (SAF ρ = 0.082, *P*_*adj*_ = 0.818; training performance ρ = − 0.246, *P*_*adj*_ = 0.168). There was an association between cardiorespiratory fitness and modulation of corticomotor excitability for PAS (ρ = 0.331, *P*_*adj*_ = 0.036; Fig. 8a), but not skill acquisition (ρ = 0.051, *P*_*adj*_ = 0.927; Fig. 8b).

Regression modelling indicated that the factor of Age (SAF t = 4.211, *P* < 0.001; Skill t = 3.082, *P* = 0.003), but not cardiorespiratory fitness (SAF t = − 0.195, *P* = 0.847; Skill t = 0.430, *P* = 0.669), was a significant predictor of baseline motor performance.

## Discussion

The present study examined M1 excitability and inhibition after PAS and motor skill acquisition in young and older adults with cardiorespiratory fitness included as a covariate. In support of our first hypothesis, skill level was greater in young compared with older adults, but skill acquisition measured as improvement in SAF and training performance did not differ between groups. There was a tendency for corticomotor excitability to increase in both young and older adults after skill acquisition, whereas corticomotor excitability increased after PAS in young but not older adults, supporting our second hypothesis. However, this finding was only evident when corticomotor excitability was normalized to baseline. Facilitation of corticomotor excitability after PAS, but not skill acquisition, was positively correlated with cardiorespiratory fitness. SICI was more effectively activated with AP stimulation than PA. Our third hypothesis was partially supported by the decreased SICI evident after PAS using AP-induced current compared to PA. In contrast, more SICI was observed after skill acquisition, and this modulation was not associated with a specific current direction. For LICI, there was an increase in young but decrease in older adults after PAS. Skill acquisition did not modulate LICI. These findings indicate that both experimentally induced and use-dependent plasticity can modulate GABAergic inhibition, although the extent of change may vary between interventions.

### Reduced motor performance in older adults

Absolute skill level was lower in older adults compared with young, corroborating previous studies (Cirillo et al. 2011; Mooney et al. 2019; Gӧkҫe et al. 2022). Consistent with the natural age-related decline in cardiorespiratory fitness (Jackson et al. 2009) and a general reduction in physical activity levels with advancing age (Hallal et al. 2012), cardiorespiratory fitness in older adults was lower than young. However, regression modelling revealed that age, not cardiorespiratory fitness, was a significant predictor for motor performance. Therefore, age-related changes in the nervous system, such as reduced brain volume and decreased cerebral gray and white matter (Courchesne et al. 2000; Jernigan et al. 2001), likely contribute to the decline in skilled motor function in older adults.

### Skill acquisition in young and older adults

Successful skill acquisition was evident for both SAF (Fujiyama et al. 2017; Mooney et al. 2019) and training performance (Cirillo et al. 2020; Durand-Ruel et al. 2023; Hand et al. 2023; Sasaki et al. 2024) in young and older adults. Older adults tend to make slower movements compared with young to maintain a similar level of accuracy (Lamb et al. 2016). Consequently, each participant completed the sequential visual isometric force task at an externally (SAF) and self-selected (training performance) pace to quantify skill acquisition. Absolute skill levels were assessed because baseline differences in performance between age groups may lead to comparisons that are conceptually fraught when using analyses of normalized data (Hardwick et al. 2017). Overall, older adults maintain the ability to acquire a novel motor skill despite exhibiting a lower absolute skill level compared with young adults.

The relationship between cardiorespiratory fitness and skill acquisition has not been clearly identified. A previous study showed that fitness levels had no effect on the improvement of mirror star trace performance in young and older adults, with a median split approach used within each age group to categorize those less and more fit (Etnier and Landers 1998). The same researchers later reported enhanced skill acquisition of the mirror star trace task in participants with higher physical fitness levels than lower (Etnier et al. 2001). However, the study by Etnier et al. (2001) did not account for age with more young adults included in the higher fit group and more older adults in the lower fit group. Subsequent literature demonstrates that better implicit motor sequence learning in older adults is linked to higher cardiovascular fitness (Zwingmann et al. 2021). The present study did not show an association between cardiorespiratory fitness and skill acquisition of a visual isometric force task, consistent with previous findings in young adults (Hung et al. 2021). One possible explanation is that participants were healthy individuals who are likely to have multiple mechanisms at their disposal for motor system function, reducing the observable impact of fitness on skill acquisition. Alternatively, the influence of cardiorespiratory fitness may be more evident in task retention rather than initial learning (Skriver et al. 2014). Further research is needed to clarify whether physical fitness influences motor learning, particularly in aging populations.

### CST activation in young and older adults

MEP onset latency was ~ 1.5 ms longer for each current direction in older adults compared with young. This delay may reflect reduced conduction velocity along the corticospinal pathway commonly observed with advancing age (Taylor 1984; Bouche et al. 1993; Metter et al. 1998). In both young and older participants, MEP onset latency was shortest using LM stimulation. Corroborating previous literature, early (PA–LM) and later onset (AP–LM) I-wave recruitment efficiency, which are independent of corticospinal conduction velocity, did not differ between age groups (Opie et al. 2018). This finding indicates that preferential activation of circuits responsible for early and later onset I-waves may not be diminished with aging, although detecting more subtle changes may require invasive methodology, such as epidural recordings.

There were no age-related differences in MEP thresholds for PA and AP stimulation, but stimulation intensity for LM to elicit a MEP was higher in older adults than young. LM stimulation preferentially activates corticospinal neurons directly (D-waves), whereas PA and AP stimulation do so indirectly from trans-synaptic activation by intracortical circuits (I-waves) (Di Lazzaro and Ziemann 2013). Consequently, LM stimulation targets deeper cortical structures than PA and AP. TMS induced electric fields are influenced by anatomical factors, with weaker fields modeled in older adults because of age-related increases in skull thickness, extra-axial space, and variation of inner tissues (i.e., gray matter, white matter, cerebrospinal fluid) (Alawi et al. 2023). While reduced corticospinal neuronal excitability with advancing age is possible, this notion in isolation seems unlikely because a higher stimulation intensity would be expected for each TMS current direction. Therefore, higher TMS intensities in older adults compared with young may be most prominent when the induced current preferentially targets neurons located in deeper cortical regions.

### PAS and neurophysiology

#### Corticomotor excitability

Modulation of the MEP, a measure of corticomotor excitability, is often used as a marker of M1 plasticity (Stefan et al. 2000; Rosenkranz and Rothwell 2006). Normalized MEP data showed increased corticomotor excitability after PAS in young, but not older, adults. This finding corroborates with those of a systematic review exploring the effect of age on non-invasive brain stimulation, indicating that responsiveness to LTP-like PAS is less in older adults than young (Shah et al. 2024). Membrane potential (i.e., RMT) and non-conditioned threshold estimate (MEP amplitude of 200 μV) were not different between age groups or modulated by PAS, but MEP onset latency was slightly delayed (~ 1.5 ms) in older adults compared with young. Based on the spike-timing dependent plasticity properties of PAS (Müller-Dahlhaus et al. 2010), ISI may affect synchronous arrival. However, previous age-related LTP-like PAS studies show diminished MEP amplitude in older adults compared with young for ISIs set to 25 ms (Tecchio et al. 2008; Fathi et al. 2010) and individualized (N20 + 2 ms) (Müller-Dahlhaus et al. 2008). Furthermore, there was no correlation between PA MEP latency and modulation of corticomotor excitability after PAS in the present study (Supplementary Table 2). It is also noted that the non-normalized analysis of corticomotor excitability did not reveal a significant effect of time or age × time interaction. This approach can be influenced by individual differences in baseline corticomotor excitability, introducing substantial variability that may obscure meaningful effects. The normalized analyses were pre-planned to account for individual differences in baseline corticomotor excitability, aimed at improving sensitivity to within-subject changes over time. While the non-significant effects of non-normalized data suggests an overall lack of systematic change across groups, the normalized results highlight a potential age-related difference in PAS responsiveness. Based on these LTP-like PAS findings, it is tempting, albeit cautiously, to infer that M1 plasticity declines with advancing age.

In the present study, cardiorespiratory fitness was lower in older adults compared with young. Regular aerobic exercise can influence induction of plasticity (El-Sayes et al. 2019b). Cardiorespiratory fitness is altered by overall physical activity and may provide a stable reflection of recent physical activity levels (Lang et al. 2018). There was a positive correlation between cardiorespiratory fitness and facilitation of corticomotor excitability, which supports previous LTP-like PAS results in young adults (Cirillo et al. 2009). However, this relationship was not present when separated by age group (Supplementary Table 1), likely due to the smaller sample size and restricted *V*O_2_ peak ranges within each group. Reduced levels of physical activity that commonly accompany advanced age may result in a less conducive environment to promote plasticity, contributing to the age-dependent reduction in LTP-like PAS. For example, animal studies show that regular exercise may improve blood flow from increased angiogenesis in M1 (Kleim et al. 2002; Swain et al. 2003) and increase neurotrophic factors (e.g., brain-derived neurotrophic factor) in M1 that facilitate the survival and differentiation of neurons (Klintsova et al. 2004; Vaynman and Gomez-Pinilla 2005). Therefore, participation in regular physical activity, or perhaps a greater prevalence of physical inactivity, may be a factor that influences variability in experimentally induced M1 plasticity.

#### Intracortical inhibition and facilitation

Reduced SICI within M1 was evident after LTP-like PAS for AP, but not PA, stimulation. Adaptive threshold-hunting did not reveal any age-related differences in inhibition, but more SICI was recorded with AP stimulation than PA, corroborating previous studies (Cirillo and Byblow 2016; Sale et al. 2016; Cirillo et al. 2018). The lack of SICI modulation after PAS for PA stimulation corresponds with previous literature (Ridding and Taylor 2001; Stefan et al. 2002; Sale et al. 2007; Cirillo et al. 2009). Contamination of SICI by facilitatory inputs to the observed results seems unlikely because the conditioning intensity for SICI was below the threshold for facilitatory contamination (Peurala et al. 2008) and SICF decreased, rather than increased, after LTP-like PAS. Preferential activation of circuitry more susceptible to SICI with an AP induced current may provide a more robust measure of inhibition than PA, whereby more subtle changes in GABA_A_ receptor-mediated inhibition, such as those resulting from LTP-like PAS, can be detected.

The reduction in SICI with AP stimulation was not age-dependent, whereas corticomotor excitability (normalized to baseline) increased in young but not older adults when assessed with PA stimulation. Several factors may explain why similar age-related changes were not observed across these TMS measures. For example, corticomotor excitability reflects the overall corticospinal connection, whereas SICI specifically probes a subset of interneurons projecting onto corticospinal tract neurons (Spampinato et al. 2023). Additionally, descending volleys preferentially elicited by PA and AP stimulation differ, indicating that each current direction likely engages different sites of the same cortical element and/or different populations of cortical neurons (Di Lazzaro et al. 2001). Given these differences, it is perhaps unsurprising that age-related changes were not evident in both corticomotor excitability and SICI.

Modulation of LICI within M1 after PAS was age-dependent, with an increase in young adults and decrease in older adults. A decrease in LICI after LTP-like PAS has been reported in healthy participants (Russmann et al. 2009; Meunier et al. 2012), particularly older adults (Lu et al. 2016). Our LICI findings in older adults align with previous literature, but differences in study design may explain contrasting results in young adults. In previous studies healthy participants included both young and older adults (Russmann et al. 2009; Meunier et al. 2012), and modulation of LICI by LTP-like PAS was most pronounced after 30 min (Meunier et al. 2012), a time point later than that assessed in the present study. Furthermore, a decrease in LICI was observed after LTP-like PAS when using a smaller 0.5 mV MEP amplitude (Russmann et al. 2009; Meunier et al. 2012), but not with the larger 1 mV MEP amplitude (Meunier et al. 2012) as used in this study. Interestingly, asymmetry in LICI using an ISI of 100 ms, with more inhibition in the dominant hemisphere than nondominant, is observed in young but not older adults (Vallence et al. 2017). More symmetrical brain activation is typically detected in healthy older adults than young during unimanual movement tasks (Seidler et al. 2010). This less focal pattern of activation in older adults may reflect compensatory mechanisms for age-related changes (Mattay et al. 2002; Heuninckx et al. 2008), although it may also be a detrimental dedifferentiation process (Carp et al. 2011). The increased LICI in young adults and its decrease in older adults after LTP-like PAS suggests an age-related alteration in GABA_B_ receptor-mediated modulation.

### Skill acquisition and neurophysiology

#### Corticomotor excitability

Facilitation of corticomotor excitability after skill acquisition was evident from the pre-planned normalized MEP data comparisons and reduced TMS intensity to elicit the non-conditioned threshold estimate. However, there was no age-related difference in corticomotor excitability and no modulation of membrane potential (i.e., RMT), coinciding with previous literature (Cirillo et al. 2011; Berghuis et al. 2016; Mooney et al. 2019). The increased MEP amplitude after skill acquisition may reflect LTP-like mechanisms (Bütefisch et al. 2000; Muellbacher et al. 2002; Sawaki et al. 2002), although facilitation of corticomotor excitability resulting from the motor execution component of the task cannot be discounted (Spampinato and Celnik 2017).

Modulation of corticomotor excitability at an individual level did not reflect the magnitude of skill improvement, which is consistent with previous studies (Cirillo et al. 2011, 2012, 2020; Li Voti et al. 2011). An association between MEP amplitude and task improvement may be confounded by factors influencing the plasticity response but not skill acquisition, such as attentional demand required for the task, history of synaptic activity, and genetic effects (see Ridding and Ziemann 2010). Furthermore, the sequential visual isometric task relies on complex visuomotor processing across a distributed network of brain regions (Krakauer et al. 2019) and multiple circuits that contribute to the MEP may have no or different implications on motor performance after skill acquisition (Bestmann and Krakauer 2015). Therefore, it may not be surprising that MEP amplitude and behavior are not bijective.

Cardiorespiratory fitness was not correlated with facilitation of corticomotor excitability. Long-term aerobic exercise modulates several neural mechanisms across molecular, cellular, structural, and functional levels, which can benefit motor function and learning (El-Sayes et al. 2019a). However, only a few studies have examined whether regular aerobic exercise influences corticomotor excitability. Increased strength of corticomotor connections, determined from MEP recruitment curves, has been reported in physically active young adults (Cirillo et al. 2009). Subsequent studies have recruited untrained or sedentary young participants to undergo an aerobic exercise intervention. The MEP amplitude elicited at a single stimulation intensity increased after 12-weeks of moderate intensity aerobic training (Roeh et al. 2018; Moscatelli et al. 2020). In contrast, no modulation of MEP recruitment curves was observed after 6 weeks of high-intensity interval training (Nicolini et al. 2019). Whether discrepancies between intervention studies stem from differences in stimulation parameters, training duration, or exercise intensity remains unclear. While regular physical activity appears to positively affect M1 function in young adults, the underlying mechanisms and whether they are influenced by healthy aging remain to be elucidated.

#### Intracortical inhibition and facilitation

Increased corticomotor excitability after skill acquisition was accompanied by increased SICI within M1. Previous studies show that reduced SICI ensues sequential visual isometric force task training (Coxon et al. 2014; Mooney et al. 2019), which may promote M1 plasticity (Ziemann et al. 2001) and improve task performance (Liepert et al. 1998; Garry et al. 2004; Perez et al. 2004; Garry and Thomson 2009; Cirillo et al. 2011). However, a downregulation of phasic GABAergic inhibition is most evident during the early stages of skill acquisition (Coxon et al. 2014) and is not a consistent finding (Rosenkranz and Rothwell 2006; Rogasch et al. 2009; Cirillo et al. 2010; Ho et al. 2022). The timing of GABA_A_ receptor function assessment in human M1 may influence the detection of skill acquisition-related disinhibition.

The increased SICI after skill acquisition in the present study corroborates our previous finding (Cirillo et al. 2020). Despite a shorter training period than previous, sequential visual isometric task performance plateaued before the final block. It is plausible that once performance has plateaued there is an upregulation of GABAergic inhibition that may promote consolidation of the newly acquired skill (Shibata et al. 2017). Increased inhibition after the sequential visual isometric task may also reflect homeostatic mechanisms that maintain a balance between excitation and inhibition to prevent excessive excitability and allow normal function of cortical circuits (Kaleb et al. 2021). While more inhibition was evident with AP stimulation than PA, as expected (e.g., Cirillo and Byblow 2016), increased SICI after skill acquisition was not current direction specific or different between young and older adults. Therefore, it remains to be elucidated whether upregulation of GABAergic inhibition during skill acquisition is age dependent.

There was no modulation of LICI after skill acquisition. This finding coincides with a previous study using adaptive threshold-hunting paired-pulse TMS to quantify the extent of LICI with an ISI of 100 ms (Mooney et al. 2019). However, decreased LICI after skill acquisition was observed with an ISI of 150 ms (Mooney et al. 2019). Additionally, LICI at an ISI of 150 ms may be reduced in older adults compared with young (Opie and Semmler 2014). Future age-related studies may wish to investigate the duration rather than magnitude of postsynaptic GABA_B_ receptor after skill acquisition.

There was no age-related difference for SICF targeting the early I-wave or modulation after skill acquisition, corroborating previous sequential visual isometric task literature (Mooney et al. 2019). Age-related changes in I-wave characteristics assessed through SICF have been observed, particularly temporal characteristics associated to late I-waves (Opie et al. 2018). In addition, modulation of SICF after skill acquisition in young adults was evident for late I-waves, rather than the early I-wave (Ho et al. 2022). Whether neuronal circuits responsible for generating later onset I-waves contribute to skill acquisition in young and older adults warrants further investigation.

##### Corticomotor excitability after PAS and sequential visual isometric task

There was no association between corticomotor excitability facilitation after PAS and skill acquisition. Neural pathways involved in motor skill acquisition and those activated with PAS involve overlapping and functionally important cortical circuits (Ziemann et al. 2004; Stefan et al. 2006; Jung and Ziemann 2009; Rajji et al. 2011). However, this relationship is likely to be complex because PAS targets only a subset of synapses involved in encoding the motor task. Therefore, interpretation of MEP amplitude changes and their functional significance remains unclear.

##### Limitations

The current study has some limitations. First, this study was not a clinical trial and used an open-label design (i.e., participants and experimenters were aware of the assigned intervention). Future studies may consider a blinded approach, although this also presents challenges. For example, repeated peripheral nerve stimulation at the wrist that preceded TMS over M1 by an interval of 100 ms did not modulate MEPs (Stefan et al. 2000) and has been used as a control protocol (Jung and Ziemann 2009; Kang et al. 2011; Hamada et al. 2014). However, a potential drawback of this method is that repeated low frequency (0.25 Hz) single-pulse TMS can increase corticomotor excitability (Pellicciari et al. 2016). Full blinding is also difficult in studies of motor skill acquisition, but including a non-skilled acquisition session where no task dynamics and kinematics are learnt (Spampinato and Celnik 2017; Mooney et al. 2021; Ho et al. 2022) may help disentangle whether changes in neurophysiology are task specific. Second, our paired-pulse TMS measures were limited to a single CS intensity. Assessing paradigms such as SICI across multiple CS intensities (e.g., 70, 80, 90% AMT) allows derivation of broader indices (e.g., area under the curve, recruitment slope) that more comprehensively reflect inhibitory network dynamics. While modulation of intracortical inhibition was observed at a single CS intensity in this study, future work may benefit from using a range of stimulation parameters. Third, the focus was on skill acquisition without assessing task retention. Previous literature indicates that improvements in task retention are a consistent outcome when examining exercise and motor training (Skriver et al. 2014), suggesting retention may be a more salient behavioral measure when assessing cardiorespiratory fitness. In addition, our sample size is consistent with recent M1 plasticity studies using TMS in young and older adults (e.g., Hand et al. 2023), but larger samples are likely needed to capture the full range of cardiorespiratory fitness across age groups. Fourth, while habitual physical activity is the primary means of improving cardiorespiratory fitness, other factors such as genetics also contribute (Williams et al. 2017). Physical activity levels can be assessed subjectively (e.g., self-reported questionnaires) and objectively (e.g., accelerometry). Despite their limitations (Skender et al. 2016), these methods can offer insights into sedentary behavior (Marconcin et al. 2021). While unpacking how regular physical activity or sedentary behavior influences M1 plasticity is beyond the scope of the current study, further investigation that uses both physical activity and cardiorespiratory fitness is warranted.

In summary, behavioral findings show that skill level was greater in young compared with older adults, but skill acquisition did not differ between groups. Neurophysiology findings show that modulation of GABAergic inhibition is influenced by the type of intervention and LICI, which is thought to reflect GABA_B_ receptor-mediated inhibition, may also be modulated differently between healthy young and older adults. Corticomotor excitability normalized to baseline values tended to increase after skill acquisition that was not age-dependent, whereas an increase after PAS was demonstrated in young but not older adults. For PAS, higher cardiorespiratory fitness was associated with greater corticomotor excitability facilitation, indicating that regular physical activity can influence experimentally induced M1 plasticity and may account for some inter-individual variability in plasticity responses. Insights from this study contribute to our understanding of possible age-related declines in brain and motor-cognitive function, which may help inform prospects for health gain in older adults.

## Supplementary Information

Below is the link to the electronic supplementary material.

Supplementary file1 (DOCX 32 KB)

## Data Availability

Data from this study will be made available upon reasonable request to the corresponding author.

## Abbreviations

AMT: Active motor threshold
EMG: Electromyography
FDI: First dorsal interosseous muscle
GABA: Gamma aminobutyric acid
LTP: Long-term potentiation
M1: Primary motor cortex
MEP: Motor evoked potential
MSO: Maximum stimulator output
PEST: Parameter estimation by sequential testing
RMT: Rest motor threshold
TMS: Transcranial magnetic stimulation
CS: Conditioning stimulus
TS: Test stimulus
ISI: Interstimulus interval
SICI: Short-interval intracortical inhibition
LICI: Long-interval intracortical inhibition
SICF: Short-interval intracortical facilitation
PAS: Paired associative stimulation
LM: Lateromedial
PA: Posterior–anterior
AP: Anterior–posterior
MVC: Maximum voluntary contraction
SAF: Speed accuracy function
*V*O_2_ peak: Maximal rate of oxygen consumption
BP: Blood pressure
HR: Heart rate
ECG: Electrocardiogram
RPE: Rating of perceived exertion
