# Supplementary material for: Modulation of primary motor cortex after experimentally induced and use-dependent plasticity in young and older adults
Source: Exp Brain Res. 2025 May 30;243(7):159. doi: 10.1007/s00221-025-07107-7 (PMC12125097; doi:10.1007/s00221-025-07107-7)
Supplement: Supplementary file 1 — Supplementary file1 (DOCX 32 KB) [file 221_2025_7107_MOESM1_ESM.docx]

**Supplementary Table 1. Spearman correlation analyses for each age group**

|  |  | ρ |  | P-value* |
| --- | --- | --- | --- | --- |
| *Modulation of corticomotor excitability after PAS and after skill acquisition* |  |  |  |  |
| Young |  | 0.317 |  | 0.173 |
| Older |  | 0.228 |  | 0.233 |
| *SAF and modulation of corticomotor excitability after skill acquisition* |  |  |  |  |
| Young |  | -0.039 |  | 0.871 |
| Older |  | -0.313 |  | 0.098 |
| *SAF and cardiorespiratory fitness* |  |  |  |  |
| Young |  | 0.089 |  | 0.710 |
| Older |  | 0.101 |  | 0.599 |
| *Training performance and modulation of corticomotor excitability after skill acquisition* |  |  |  |  |
| Young |  | 0.311 |  | 0.181 |
| Older |  | 0.048 |  | 0.805 |
| *Training performance and cardiorespiratory fitness* |  |  |  |  |
| Young |  | -0.247 |  | 0.293 |
| Older |  | -0.340 |  | 0.072 |
| *Cardiorespiratory fitness and modulation of corticomotor excitability after PAS* |  |  |  |  |
| Young |  | 0.257 |  | 0.273 |
| Older |  | 0.297 |  | 0.105 |
| *Cardiorespiratory fitness and modulation of corticomotor excitability after skill acquisition* |  |  |  |  |
| Young |  | 0.206 |  | 0.382 |
| Older |  | 0.058 |  | 0.766 |

*Note that P-value has not been corrected for multiple comparisons

**Supplementary Table 2. Spearman correlation analyses: Modulation of corticomotor excitability after PAS and PA MEP latency**

|  |  | ρ |  | P-value* |
| --- | --- | --- | --- | --- |
| All Participants |  | -0.039 |  | 0.786 |
| Young |  | 0.042 |  | 0.861 |
| Older |  | 0.088 |  | 0.643 |

*Note that P-value has not been corrected for multiple comparisons
